# WNT8B as an Independent Prognostic Marker for Nasopharyngeal Carcinoma

**DOI:** 10.3390/curroncol28040230

**Published:** 2021-07-08

**Authors:** Chawalit Ngernsombat, Pongphol Prattapong, Noppadol Larbcharoensub, Krittika Khotthong, Tavan Janvilisri

**Affiliations:** 1 Graduate Program in Molecular Medicine, Faculty of Science, Mahidol University, Bangkok 10400, Thailand; chawalit.ngr@student.mahidol.ac.th (C.N.); pongphol.pra@student.mahidol.ac.th (P.P.); 2 Department of Pathology, Faculty of Medicine Ramathibodi Hospital Mahidol University, Bangkok 10400, Thailand; noppadol.lar@mahidol.ac.th; 3 N Health Pathology Laboratory, Bangkok 10310, Thailand; krittika.kho@student.mahidol.ac.th; 4 Department of Biochemistry, Faculty of Science, Mahidol University, Bangkok 10400, Thailand

**Keywords:** WNT8B, nasopharyngeal carcinoma, Wnt signaling, prognosis

## Abstract

Background: Members of the Wnt signaling pathway have been shown to play a role in nasopharyngeal carcinoma (NPC) progression. Aim: The purpose of this study was to investigate WNT8B protein expression in NPC patients using tissue microarray (TMA) analysis and to evaluate its correlation with patient survival and clinical parameters. Methods: A total of 82 NPC cases, together with six normal nasopharyngeal tissue samples, were targeted to construct the TMA blocks. The WNT8B protein expression was evaluated by immunohistochemistry and its correlation to the clinicopathological features was investigated. Results: Sixty-two of 82 (75.6%) cases exhibited high WNT8B protein expression while 20/82 (24.4%) cases appeared to have low WNT8B expression. The univariate analysis revealed that systemic metastasis was associated with patient 5-year survival. The multivariate Cox proportional hazard regression analysis showed that WNT8B expression and systemic metastasis were significantly associated with the survival of NPC patients. Furthermore, there was no correlation found between the WNT8B protein expression and other clinicopathological parameters. Conclusion: Our results suggest that the expression of WNT8B is associated with NPC patients’ survival and could serve as an independent prognostic factor for NPC patients.

## 1. Introduction

Nasopharyngeal carcinoma (NPC) is a primary malignancy arising from the epithelial cells of the nasopharynx. The tendency of the NPC incidence rate is specific to certain ethnic and geographic populations, especially in Southern China and Southeast Asia including Thailand [1,2]. A number of factors play a role in the onset of disease, such as salted-preserved food consumption [3], alcohol consumption, tobacco smoking [4], and Epstein–Barr virus (EBV) infection, and they also contribute to the progression of NPC [5,6,7]. NPC treatment is usually initiated by radiation, which is effective at the early stage [8]. However, due to its anatomical location, the symptoms of NPC are often misdiagnosed; hence, patients are often present in the late stages of NPC, leading to poor prognosis. In advanced NPC, radiotherapy alone is not effective; thus, concurrent chemotherapy is usually applied to improve patient outcomes [9]. Thus, potential molecular biomarkers for NPC prognosis need to be explored.

Wnt signaling, first discovered in the developmental processes of *Drosophila melanogaster* [10], is a signal transduction pathway that regulates various cellular processes and plays a key role in the gastrulation process of mammalian development [11]. The Wnt signaling pathway can be divided into β-catenin-dependent (canonical) signaling, in which β-catenin translocates to the nucleus and upregulates oncogenes, and β-catenin-independent (non-canonical) signaling. Both canonical and non-canonical pathways require the binding of Wnt ligands to the corresponding membrane receptor Frizzled (Fzd) proteins to activate the signal cascades. Wnt signaling has been shown to be implicated in the development and progression of many types of cancer, including gastric cancer, lung cancer, colorectal cancer, leukemia, bladder cancer, and NPC [12,13,14,15,16,17,18,19]. In NPC, certain members of the Wnt signaling pathway, such as Fzd, Wnt ligands, and β-catenin, have been demonstrated to be aberrantly expressed [15,17], thereby promoting the progression and aggressiveness of NPC. The human WNT8B was first sequenced and characterized in 1996 and was found in various tissues, including in fetal and adult stages [20]. WNT8B plays an important role in the development of vertebrates; its transcript was found during the late gastrulation process [21] and found to repress the early eye and retinal progenitor cell formation in zebrafish via the WNT/β-catenin pathway [22]. Recently, WNT8B has been observed to be involved in the development of phosphate-induced vascular smooth muscle cell calcification in chronic kidney disease through the WNT/β-catenin signaling pathway [23]. Wnt8B gene expression has been shown to be upregulated in gastric cancer cells, breast cancer cells, and embryonal tumor cells [24,25]. Wnt8B then plays a potential role in cancer progression through the canonical Wnt signaling pathway. Herein, we therefore investigated the expression of the WNT8B protein in 82 NPC tissue samples using tissue microarray (TMA) and immunohistochemistry. The association between the expression of WNT8B, clinicopathological features, and patient outcome was also elucidated.

## 2. Materials and Methods

### 2.1. Patients and Tissue Specimens

A total of 82 formalin-fixed, paraffin-embedded (FFPE) tissue samples of NPC patients were collected from Ramathibodi Hospital between 2000 and 2008 (with a median age of 50 years old, ranging from 16 to 79 years old). All specimens were taken at the time of diagnosis of NPC, prior to therapy. Clinicopathological characteristics of the patients are presented in Table 1. TNM stages and histological grades were evaluated according to the 6th edition of the American Joint Committee on Cancer (AJCC) staging system and the World Health Organization (WHO) classification, respectively. All patients were treated with radiation to the primary tumor at 18 to 20 Gy daily, 5 times/week, with a total dose ~70 Gy. Concurrent chemotherapy with cisplatin at the dose of 100 mg/m^2^ was given during weeks 1, 4, 7, 10, 13, and 16 of radiotherapy. The patients then received chemotherapy consisting of cisplatin (80 mg/m^2^) on day 1 and 5-fluorouracil (1000 mg/m^2^/day) during days 1 to 5 every 4 weeks for 3 cycles after the completion of concurrent chemoradiotherapy. All patients were examined routinely every 3–6 months during the first 5 years of follow-up. This study received ethics approval from the Ethics Committee of Faculty of Medicine Ramathibodi Hospital, Mahidol University.

### 2.2. Tissues Microarray Construction

NPC tissues were obtained from FFPE specimens, and 2.0 mm cores were arrayed in a recipient block in TMA format. The tumor areas taken for the TMA construction were labeled on hematoxylin and eosin-stained slides and the donor block by a pathologist. According to the various sizes of the tissues, two or three tissue cores with a 2 mm diameter and 3–4 mm height of tumor area were taken from each donor tumor block, and the distance between each selected tissue core was at least 1 mm [26,27]. The tissue cores were placed into a recipient paraffin block that contained 60 cores for tissue insertion with Arraymold Manual Tissue Microarrayer (IHCworld) and melted in a paraffin acceptor block. Six normal nasopharyngeal and normal tissues from colon, lung, thyroid, kidney, and liver were also inserted into the TMA. The independent blocks of this TMA were analyzed and averaged.

### 2.3. Immunohistochemistry

The tissue microarray blocks were sectioned into pieces of 5 μm thickness and subjected to immunohistochemistry. The tissue microarray sections were deparaffinized by soaking in xylene for 3 min 2 times and rehydrated by a serial dilution of ethanol: absolute ethanol, 95%, 85%, 70%, 50%, 30%, and type II H_2_O for 3 min each. Ten millimolar of sodium citrate pH 6.0 was used for antigen retrieval at 95 °C for 10 min. The sections were blocked with bovine serum albumin for 30 min, washed with phosphate-buffered saline (PBS), and incubated with rabbit anti-Wnt8B antibody (1:200 dilution; catalog number AAS91334E, Antibody Verify, Las Vegas, NV 89148, USA) at 4 °C overnight in a humidified chamber. The antibody was replaced by IgG1 for negative controls. Then, the microarray tissue sections were washed 3 times with PBS and further subjected to SignalStain^®^ Boost IHC Detection Reagent (Cell Signaling Technology, Danvers, MA 01923, USA) for 1 h for 30 min at room temperature. The target protein was visualized with 3-amino-9-ethylcarbazole peroxidase substrate containing H_2_O_2_ for 20 min. The microarray tissue sections were then washed with PBS and counter-stained using hematoxylin. The protein expression and localization in each tissue sample was observed by visualization under a BX53 Olympus microscope.

### 2.4. IHC Scoring

The quantitation of immunostaining for WNT8B was evaluated based on the intensity and heterogeneity by two independent pathologists, who were blinded regarding patient details. Where there were 3 disagreement cases among study pathologists, a consensus was reached in all cases following discussion. The estimated agreement was 96.3% and the Cohen’s kappa coefficient was equal to 0.907, with a 95% confidence interval of 0.8212–0.9924. Positive staining indicated the cytoplasmic reaction of the epithelial cells, and the lymphocytes were excluded from the interpretation. Sections with no WNT8B labeling were scored as 0, labeling of >0–25% cells was scored as 1, labeling of 26–50% cells was scored as 2, labeling of 51–75% of cells was scored as 3, and labeling of 76–100% of the cells was scored as 4. The staining intensity of positive cells was scored as 0 for negative, 1 for weakly positive, 2 for moderately positive, and 3 for strongly positive. The product was calculated using the formula: labeling score × intensity scores. Low and high WNT8B expression score was defined according to Log-rank statistical analysis with respect to the significance of NPC patient survival [28,29,30]. A final score of 1–2 indicated low expression and 3–12 indicated high expression.

### 2.5. Statistical Analysis

The significance of differences between groups was evaluated with chi-square test. Univariate analyses were performed to determine the prognostic factors associated with 5-year survival. Univariate and multivariate analyses through Cox proportional hazard regression were performed using all variables including age, gender, WHO classification, AJCC staging, TNM stage, the presence of tumor recurrence, and WNT8B expression pattern. Survival curves were plotted by the Kaplan–Meier method. Two-sided *p*-values were calculated, and a probability level < 0.05 was considered statistically significant. All statistical data were evaluated using IBM SPSS software v21.0 (IBM Corp, Armonk, NY, USA).

## 3. Results

### 3.1. WNT8B Expression in NPC and Normal Nasopharyngeal Tissues

**Figure 1:**
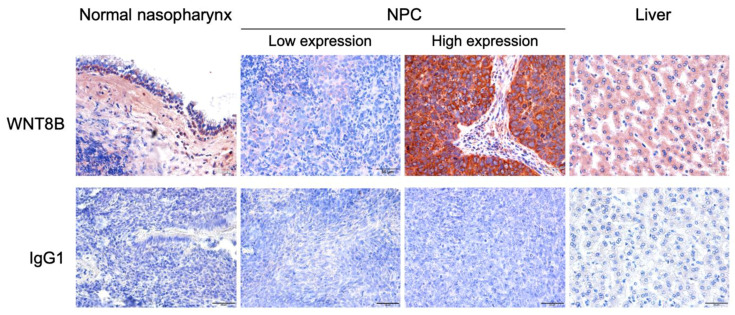
WNT8B protein expression in nasopharyngeal carcinoma. Shown are representative photomicrographs at magnification X200 of normal nasopharynx and cancerous nasopharyngeal tissues that were subjected to immunostaining of WNT8B; human liver tissues were used as positive control. The WNT8B antibody was replaced by IgG1 for the isotype negative control.

WNT8B protein expression was analyzed in 82 NPC tissue samples through immunohistochemistry. Six normal nasopharyngeal tissues were also included. The detailed patient profiles are summarized in Table 1. Representatives of cytoplasm WNT8B immunoreactivities are shown in Figure 1. The IgG1 antibody was included as an isotypic negative control. The results revealed that normal nasopharyngeal tissues expressed WNT8B comparably to liver tissue, which was reported to indicate low WNT8B expression [31]. Among NPC samples, the IHC scores based on WNT8B expression were used to categorize the patients into two groups: 20 and 62 patients with low and high WNT8B expression, respectively.

### 3.2. Correlation of WNT8B and Clinicopathological Features

The association between NPC patient survival and clinicopathological characteristics including age, gender, WHO classification, AJCC stage, T classification, regional lymph node metastasis, systemic metastasis, recurrence, and WNT8B expression was evaluated (Table 2). The univariate analysis revealed that regional lymph node metastasis and systemic metastasis were correlated with 5-year survival, with *p*-values of 0.048 and 0.003, respectively. Furthermore, univariate analysis also revealed that systemic metastasis was associated with survival time (*p* < 0.001). The independency tests were carried out to evaluate the prognostic indicator through multivariate Cox regression analysis. Our results showed that high WNT8B expression was an independent prognostic factor for poor NPC patient survival, with a relative risk of death of 1.744 (95% confidence interval: 1.005–3.025, *p*-value = 0.048), among another established prognostic risk factor, systemic metastasis (*p* < 0.001).

**Figure 2:**
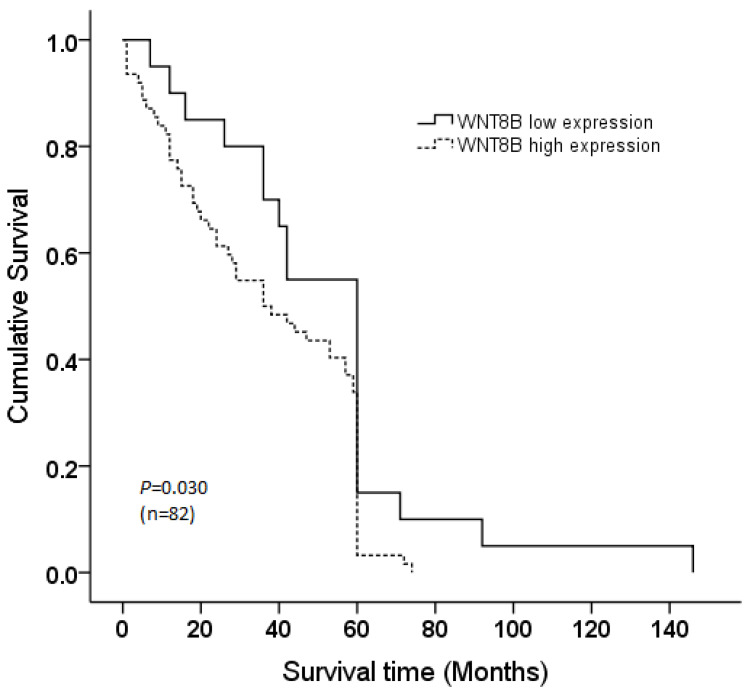
Kaplan–Meier survival curve of NPC patients according to WNT8B expression. The cumulative survival plot stratified by the level of WNT8B expression showed Log-rank statistically significantly correlated with NPC survival (*p*-value = 0.030).

The Kaplan–Meier analysis illustrated a significant correlation of WNT8B expression and NPC patient survival, as shown in Figure 2. NPC patients with high expression of WNT8B had significantly shorter survival times, with a mean survival time of 37.0 ± 3 months, compared to those with a low level of WNT8B expression, with a mean survival time of 52.3 ± 7 months. However, there was no statistically significant correlation between WNT8B expression and other clinicopathological features (Table 3), which confirmed the independence of WNT8B expression from other clinical parameters.

## 4. Discussion

Wnt signaling is a transduction pathway that plays a key role in the developmental process of mammalian cells, including proliferation, differentiation during both embryogenesis and adult cell development, and stem cell renewal and maintenance [32]. Wnt signaling is important for maintaining a balance between self-renewal and differentiation in cells, which maintains stem cells and regulates cell fate decisions [33,34]. The Wnt signaling pathway is tightly related the cancer [35] and its dysregulation is important for the carcinogenesis of various types of cancer, including colorectal cancer, lung cancer, breast cancer, and head and neck squamous cell carcinomas [36]. Several studies showed the involvement of the canonical WNT protein in the WNT/β-catenin signaling pathway and cancer progression. For example, the positive expression of WNT1 was correlated with a short overall survival time and involved in lung cancer and colorectal cancer metastasis [37,38,39,40]. WNT2 expression has been significantly associated with cancer development in pancreatic cancer, gastric cancer, colorectal cancer, cervical carcinoma, and non-small cell lung cancer [41,42,43,44,45]. WNT3A regulates the cell cycle and metastasis and acts as a prognostic marker of hepatocellular carcinoma [46,47,48]. In head and neck cancer, the level of WNT3A was increased in patients’ serum, which enhanced radioresistance by WNT/β-catenin pathway activation in NPC cells [49], and it may represent an independent prognostic factor in laryngeal squamous cell carcinoma [50]. Thus, aberrant expression of canonical WNT ligands tends to promote cancer aggressiveness, including enhanced cancer cell progression, metastasis, and poor clinical outcomes.

WNT8B is one of the canonical WNT ligands whose expression has been widely identified in cancers, namely ameloblastoma, breast cancer, pancreatic cancer, brain tumors, embryonic tumors, colorectal cancer, human, and gastric cancer [24,25,51,52]. Furthermore, Wnt8B was reported to be upregulated in hepatocellular carcinoma and served as a valuable diagnostic and prognostic biomarker and a therapeutic target for hepatocellular carcinoma patients [53]. In this study, we investigated the protein expression of WNT8B in NPC. TMA revealed the differential immunoreactivity of the WNT8B protein in NPC tissues. Cox multivariate regression analysis suggested that systemic metastasis in NPC was significantly correlated with patient survival, which was supported by evidence that distant metastatic NPC reduced the patients’ survival even when radiotherapy was applied [54]. Interestingly, our study is the first report to reveal a significant correlation between WNT8B protein expression and NPC patient survival. Our results found that high WNT8B protein expression and systemic metastasis were independent biomarkers associated with poor prognosis in NPC patients. However, the mechanism of WNT8B in NPC progression needs to be further investigated. To the best of our knowledge, the exact transduction signaling mechanism of WNT8B in cancer has yet to be confirmed. Nevertheless, a study showed that Wnt8B genetically interacted with Fzd3a and is important in forebrain commissural patterning in zebrafish [55]. Thus, it is possible to speculate that WNT8B participates in the WNT/β-catenin pathway through binding with the FZD3A receptor in vertebrates.

According to the transduction cascade of canonical WNT/β-catenin signaling, the signal is activated by the binding of the WNT ligand to the cellular surface receptor (Frizzled; Fzd); it then transduces the signal, causing nuclear localization of β-catenin. β-catenin, a key component of the canonical Wnt signaling pathway, has been demonstrated to be upregulated in NPC patients, which was correlated with the patient’s survival. It has been elucidated as an independent prognostic factor [56,57,58,59] and is involved in the advanced stages of NPC [17]. β-catenin has been found to regulate oncogenes such as CYCLIN D1, PDK, MTC-1, MMP7, fibronectin, COX-2, AXIN-2, and C-MYC. Most of the downstream oncogenes have been related to NPC prognosis [18,58,60,61,62,63].

## 5. Conclusions

Our studies showed that WNT8B protein expression was significantly correlated with the survival of NPC patients. Thus, it is possible that WNT8B is involved in the progression and poor prognosis of NPC. WNT8B then could be a useful independent prognostic biomarker for overall survival. Further investigations to determine potential WNT8B-targeted therapeutics are warranted and might improve the survival outcomes of these patients.

## Figures and Tables

**Table 1:** Clinicopathological characteristics of nasopharyngeal carcinoma patients in this study.

Characteristics		No. of Patients	
**Age**			
	Mean	49.29	years
	Median	50	years
	Range	16–79	years
**Gender**			%
	Male	57	69.5
	Female	25	30.5
**WHO classification**			%
	Type 1	2	2.4
	Type 2	46	56.1
	Type 3	34	41.5
**AJCC staging**			%
	Stage I–II	12	14.6
	Stage III–IV	68	82.9
	N/A	2	2.4
**T stage**			%
	T1–T2	30	36.6
	T3–T4	51	62.2
	N/A	1	1.2
**Regional lymph node metastasis**			%
	No	21	25.6
	Yes (N1–N3)	61	74.4
**Systemic metastasis**			%
	No	69	84.1
	Yes (M1)	12	14.6
	N/A	1	1.2
**Recurrence**			%
	No	58	70.7
	Yes	24	29.3
	Total	82	100.0

**Table 2:** Univariate and multivariate analyses of prognostic variables correlated with NPC patient survival.

Prognostic Variables		No. of Patients	5-Year Survival			Survival (Months)			
			No. of Patients	%	*p* Value	Univariate		Multivariate	
						HR (95% CI)	*p* Value	HR (95% CI)	*p*-Value
**Age (years)**					0.406	1.342 (0.858 to 2.099)	0.197	1.326 (0.799 to 2.201)	0.275
	<50	44	19	43.2					
	≥50	38	13	34.2					
**Gender**					0.905	1.006 (0.626 to 1.614)	0.982	0.870 (0.525 to 1.440)	0.587
	Male	57	22	38.6					
	Female	25	10	40.0					
**WHO classification**					0.146	1.426 (0.926 to 2.196)	0.107	1.202 (0.760 to 1.903)	0.432
	Type 1	2	1	50.0					
	Type 2	46	22	47.8					
	Type 3	34	9	26.5					
**AJCC staging**					0.106	1.557 (0.820 to 2.956)	0.176	1.376 (0.477 to 3.974)	0.555
	I–II	12	7	58.3					
	III–IV	68	23	33.8					
	N/A	2	-	-					
**T classification**					0.096	1.414 (0.893 to 2.238)	0.140	1.043 (0.559 to1.943)	0.895
	T1–T2	30	15	50.0					
	T3–T4	51	16	31.4					
	N/A	1	-	-					
**Regional lymph node metastasis**					**0.048** *	1.451 (0.874 to 2.411)	0.150	1.131 (0.549 to 2.329)	0.738
	No	21	12	57.1					
	Yes (N1–N3)	61	20	32.8					
**Systemic metastasis**					**0.003** *	6.207 (2.977 to 12.939)	**0.000** *	5.852 (2.614 to 13.103)	**0.000** *
	No	69	31	44.9					
	Yes	12	0	0.0					
	N/A	1	-	-					
**Recurrence**					0.856	0.819 (0.493 to 1.360)	0.441	0.832 (0.495 to 1.397)	0.486
	No	58	23	39.7					
	Yes	24	9	37.5					
**WNT8B**					0.092	1.616 (0.951 to 2.744)	0.076	1.744 (1.005 to 3.025)	**0.048** *
	Low expression	20	11	55.0					
	High expression	62	21	33.9					

* *p* < 0.05, AJCC; American Joint Committee on Cancer.

**Table 3:** Correlation of clinicopathological parameters and WNT8B expression.

Characteristics		Case	No. of Patients (%)		*p*-Value
			Low WNT8B	High WNT8B	
**No. of patients**		82	20/82 (24.4)	62/82 (75.6)	
**Age**					0.513
	<50	44	12/20 (60.0)	32/62 (51.6)	
	≥50	38	8/20 (40.0)	30/62 (48.4)	
**Gender**					0.241
	Male	57	16/20 (80.0)	41/62 (66.1)	
	Female	25	4/20 (20.0)	21/62 (33.9)	
**WHO classification**					0.694
	Type 1	2	0/20 (0.0)	2/62 (3.2)	
	Type 2	46	11/20 (55.0)	35/62 (56.5)	
	Type 3	34	9/20 (45.0)	25/62 (40.3)	
**AJCC staging**					0.532
	I–II	12	2/20 (10.0)	10/62 (16.1)	
	III–IV	68	17/20 (85.0)	51/62 (82.3)	
	N/A	2	1/20 (5.0)	1/62 (1.6)	
**T stage**					0.395
	T1–T2	30	9/20 (45.0)	21/62 (33.9)	
	T3–T4	51	11/20 (55.0)	40/62 (64.5)	
	N/A	1	0/20 (0.0)	1/62 (1.6)	
**Regional lymph node metastasis**					0.509
	No	21	4/20 (20.0)	17/62 (27.4)	
	Yes (N1–N3)	61	16/20 (80.0)	45/62 (72.6)	
**Systemic metastasis**					0.485
	No	69	18/20 (90.0)	51/62 (82.2)	
	Yes	12	2/20 (10.0)	10/62 (16.1)	
	N/A	1	0/20 (0.0)	1/62 (1.6)	
**Recurrence**					0.517
	No	58	13/20 (65.0)	45/62 (72.6)	
	Yes	24	7/20 (35.0)	17/62 (27.4)	

## Data Availability

All data generated or analyzed during this study are included in this published article.
